# Comparison of perceived and modelled geographical access to accident and emergency departments: a cross-sectional analysis from the Caerphilly Health and Social Needs Study

**DOI:** 10.1186/1476-072X-5-16

**Published:** 2006-04-13

**Authors:** David L Fone, Stephen Christie, Nathan Lester

**Affiliations:** 1 Department of Epidemiology, Statistics and Public Health, Centre for Health Sciences Research, School of Medicine, Cardiff University, Heath Park, Cardiff, CF14 4YS, UK; 2 National Public Health Service for Wales, Mamhilad Park Estate, Pontypool, Gwent, NP4 0YP, UK; 3 Centre for Social Research and Evaluation, Ministry of Social Development, Bowen State Building, Bowen Street, PO Box 1556, Wellington, New Zealand; 4 National Public Health Service for Wales, Temple of Peace and Health, Cathays Park, Cardiff, CF10 3NW, UK

## Abstract

**Background:**

Assessment of the spatial accessibility of hospital accident and emergency departments as perceived by local residents has not previously been investigated. Perceived accessibility may affect where, when, and whether potential patients attend for treatment. Using data on 11,853 respondents to a population survey in Caerphilly county borough, Wales, UK, we present an analysis comparing the accessibility of accident and emergency departments as reported by local residents and drive-time to the nearest accident and emergency department modelled using a geographical information system (GIS).

**Results:**

Median drive-times were significantly shorter in the lowest perceived access category and longer in the best perceived access category (p < 0.001). The perceived access and GIS modelled drive-time variables were positively correlated (Spearman's rank correlation coefficient, r = 0.38, p < 0.01). The strongest correlation was found for respondents living in areas in which nearly all households had a car or van (r = 0.47, p < 0.01). Correlations were stronger among respondents reporting good access to public transport and among those reporting a recent accident and emergency attendance for injury treatment compared to other respondents. Correlation coefficients did not vary substantially by levels of household income. Drive-time, road distance and straight-line distance were highly inter-correlated and substituting road distance or straight-line distance as the GIS modelled spatial accessibility measure only marginally decreased the magnitude of the correlations between perceived and GIS modelled access.

**Conclusion:**

This study provides evidence that the accessibility of hospital-based health care services as perceived by local residents is related to measures of spatial accessibility modelled using GIS. For studies that aim to model geographical separation in a way that correlates well with the perception of local residents, there may be minimal advantage in using sophisticated measures. Straight-line distance, which can be calculated without GIS, may be as good as GIS-modelled drive-time or distance for this purpose. These findings will be of importance to health policy makers and local planners who seek to obtain local information on access to services through focussed assessments of residents' concerns over accessibility and GIS modelling.

## Introduction

Geographic access to hospital, primary and emergency care health services remains an important area for health service policy [1,2]. Many studies have investigated the spatial accessibility of health services using travel impedance models of road network travel time and travel distance using geographic information systems (GIS) [3-12]. There are several types of spatial accessibility models, using indirect measures such as population density or nearest neighbour distances and direct approaches such as travel impedance and gravity models, each of which requires certain assumptions about how patients spatially interact with health services. The relative advantages and limitations of these different approaches have been reviewed, but no consensus approach has emerged [9,13].

Some of these studies have compared the results from more than one type of spatial accessibility model of health services. In a study of geographical variation in rates of acceptance to renal replacement services in England, travel distance and time were shown to be a considerably better representation of access than the simple crow-fly distance [4,5]. In a comprehensive study comparing six measures of spatial accessibility in south-west England, stronger associations were found between crow-fly distance and travel time than between population density or nearest neighbour and travel time [9]. A study correlating straight-line distance and travel time between major road intersections, as a proxy for hospital locations in New York State, found that straight line distance is a reasonable proxy for travel time, especially with large numbers of hospitals and distances of more than 15 miles [3]. Two studies set in north-west [10] and south-west England [12] also found that straight-line distance and travel time measures of accessibility of health services were highly correlated.

However, researchers have largely overlooked the role that community and patient perceptions of spatial accessibility might play in translating accessibility (potential access) into utilisation (realised access) of health services. Perception of accessibility is important because it might affect where, when and even whether patients seek or receive health care. Accessibility has been shown to influence whether patients attend for discretionary treatments. For example, a study of child utilisation of Accident & Emergency (A&E) departments found a distance decay effect for all injuries, in which there will be discretion about the need for treatment. No distance decay effect was found for severe injuries, indicated by fractures, which are considered non-discretionary [14]. In cases where the decision to attend is made by the patient, it is likely that utilisation is more strongly influenced by accessibility as perceived by the patient than by accessibility as modelled by any of the GIS-based approaches. For all conditions, including non-discretionary ones, in situations where there are multiple service locations, choice of where to attend may depend on perceived accessibility of competing locations. In conditions of gradual onset of symptoms or gradual progression of severity, perceived accessibility might also influence how long patients wait before seeking treatment. Studies of primary care accessibility found that the perception of accessibility influences the choice of services in an emergency and that people without a car are more likely than others to perceive that the nearest major A&E unit is too far away [15]. Perceived inaccessibility of primary care providers is among the leading reasons for non-urgent use of emergency departments [16].

In this paper, we aim to investigate the extent to which perceived accessibility of local A&E departments is correlated with GIS-modelled accessibility measures. The specific objectives of the study were, firstly, to investigate whether the strength of correlation between perceived and modelled accessibility was related to age, gender, household income, car ownership, perceived public transport access, and recent utilisation of A&E health services for injury treatment, and secondly, whether the strength of correlation was related to the method used to measure geographical accessibility.

**Figure 1:**
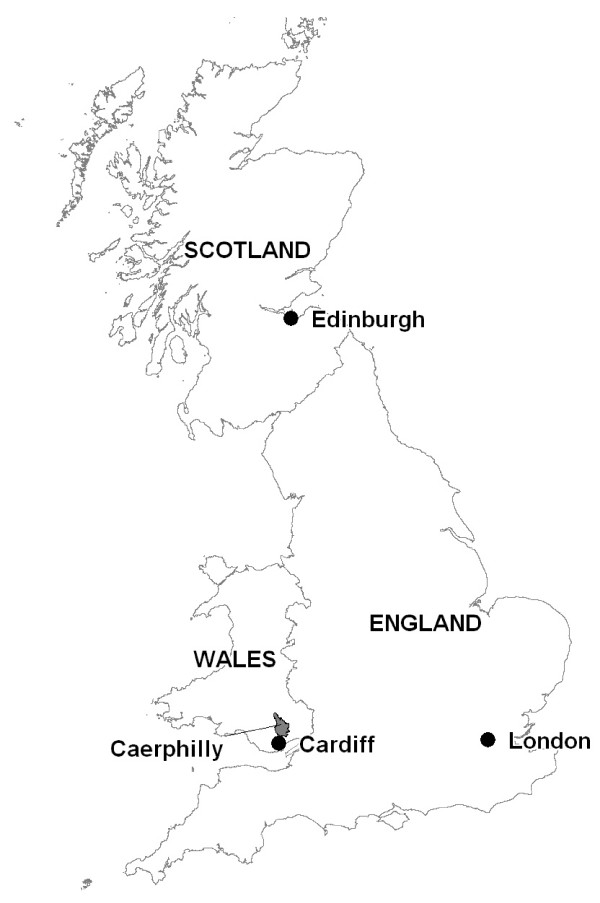
**Map of UK to show location of Caerphilly county borough in Wales**. This map is reproduced from Ordnance Survey material with the permission of Ordnance Survey on behalf of the Controller of Her Majesty's Stationery Office (c) Crown copyright. Unauthorised reproduction infringes Crown copyright and may lead to prosecution or civil proceedings. National Public Health Service for Wales, licence no. CGP0138.

The study area was Caerphilly county borough, Wales, UK, one of the 22 local government areas in Wales, UK, created in 1996 as part of the reorganisation of local government (Figure 1). We have gathered population survey data on a wide range of socio-demographic and neighbourhood factors, including perceived accessibility of A&E departments, as part of the Caerphilly Health and Social Needs Study, a population based study of health inequality in Caerphilly county borough [17]. The borough occupies 28,000 hectares of the south Wales valleys with a declining and ageing population of 169,519 (2001 Census). It stretches over 40 km between the urban centres of Cardiff and Newport in the south and the Brecon Beacons to the north. A long period of social and economic decline resulting from the closure of the traditional heavy industries of coal and steel has left the borough with some of the most deprived electoral wards in Wales and England [18]. However, unemployment in the borough is now falling and a younger, more mobile and affluent commuter population is developing in the less deprived areas of the south of the borough, bordering the outskirts of Cardiff.

## Methods

### Questionnaire survey

We carried out a postal questionnaire survey sampled from the 132,000 adult population aged 18 years and over resident in Caerphilly county borough in the autumn of 2001. The survey was granted ethical approval by Gwent Local Research Ethics Committee. The sample was drawn from the general practitioner age-sex register held by the former Gwent Health Authority and stratified by census ward. The sample size calculation was based on the objectives of the wider study of health and social inequality that required ward prevalence estimates of a range of socioeconomic factors to within ± 5 percent with 95 percent confidence. The mean required sample size was 350 in each of the 36 wards in the borough, giving a target of 12,600 responses. We aimed to achieve a 60% response and so the number of people sampled in each ward was increased to give a total of 22,290. The sample size was increased to 22,290 to allow for an anticipated 60 percent response. Of the 22,236 questionnaires posted, 12,408 were returned, equating to an adjusted response of 62.7 percent after removal of incorrect addresses.

The survey included the following questions on accessibility: 'How well placed do you think your home is for the nearest hospital with a casualty department?' and 'How well placed do you think your home is for public transport (buses, trains)?' Answers were in the form of a Likert scale with five response categories: 'Very well placed'; 'Fairly well placed'; 'Average'; Not very well placed'; and 'Not at all well placed'. Recent utilisation of A&E health services was assessed with the yes/no question: 'Have you had an accident, injury, or poisoning needing hospital treatment or a visit to Casualty in the past three months?'

The survey also asked a range of demographic and socioeconomic questions. Respondents were asked to report their gross household income in one of three bands of 'high', greater than £215 per week, 'medium', between £95 and £215 per week, and 'low', less than £95 per week. Both the 'medium' and 'low' categories are classified as 'low income' under the UK definition of a gross household income of less than 60% of median income, after housing costs [19,20]. There were 559 census output areas (COA) in Caerphilly county borough defined by the 2001 Census, with an average resident population size of 303 persons. The survey did not include a question on car ownership and so we obtained COA-level data on the proportion of households with no car or van.

### Spatial analysis

We used Mapinfo Drive-time Version 6.1 to estimate the time and distance (road length and straight-line) needed to travel from the population-weighted centroid of each respondent's COA of residence to the nearest of the six A&E departments in the area serving the study population (Caerphilly Miners Hospital; University Hospital of Wales, Cardiff; Royal Gwent Hospital, Newport; Nevill Hall Hospital, Abergavenny; Prince Charles Hospital, Merthyr Tydfil; Royal Glamorgan Hospital, Talbot Green). We assumed that the drive-time and distance were the same for each survey respondent living within each COA. Drive-times, which were based on the default travel speeds provided with the software (Table 1), ranged from one to 24 minutes. We created an interpolated surface of perceived accessibility using Vertical Mapper Version 3.0 software. The interpolation was based on finding a combination of parameters that produced a meaningful pattern from around 12 thousand points, avoiding a surface that appears random (since there are high and low responses in every location) and a uniform flat surface from oversmoothing. We therefore used an inverse distance weighting with the following parameters: grid cell size = 100 m, maximum search radius = 3 km, exponent = 2, and the maximum number of points used to calculate each grid cell value = 500. We created contour maps of both the resulting interpolated surface for the five categories of perceived accessibility, and of drive-time in five minute bands and also a smoothed overlay map which allows a quantitative assessment of the differences between residents' perceived access and GIS modelled travel time. Drive-times were range-standardised to a range of 1 to 5 and a new variable was computed to equal perceived access minus the standardised drive-time. Thus a positive difference represented worse level of perception than drive-time and a negative difference a better level of perception than drive-time. The map of the variable for the differences between perceived access and GIS modelled drive-time was smoothed using the same parameters as for the interpolated surface of perceived accessibility.

**Table 1:** Vehicle speed in miles per hour, by road type and setting

**Road type and mode of travel**	**Rural**	**Urban**	**Inner urban**	**Conurbation central core**
Motorway	65	53	43	37
A-road dual c/w	53	28	22	19
A-road single c/w	40	25	22	19
B-road dual c/w	43	22	19	16
B-road single c/w	34	19	16	12
				
Primary road dual c/w	56	31	25	22
Primary road single c/w	43	25	19	16
Private road	28	19	16	12
Unclassified road	28	19	16	12

This table is a summary of the matrix of travel speeds incorporated within the Mapinfo Drivetime software. The full matrix includes travel speeds for passing places, ferry routes, tunnels, and central London.

### Statistical analysis

We used box plots to describe the distribution of drive-times for respondents within the five perceived access groups. To determine whether drive-time varied significantly between the groups we performed Kruskal-Wallis one-way analysis of variance with drive-time as the dependent variable and perceived access as the grouping variable. We calculated Spearman's rank correlation coefficients, r, for the association between the perceived accessibility variable and the modelled drive-time variable. Perception and actual accessibility might vary by socioeconomic factors and treatment attendance. In particular, perceptions and actual travel experiences for patients without cars may be substantially different to those of patients with cars. We therefore repeated the analysis within quintiles of COA-level proportion of households without cars, which ranged from zero to 76 percent in the study area, the three categories of self-reported household income, injury status (yes/no), and the five-point Likert scale of levels of perceived public transport access. We repeated the overall correlation coefficient calculation with drive-time replaced by two other common measures of geographical separation, firstly road travel distance and secondly straight-line distance, so that we could assess whether the results were sensitive to the type of measure used. We also calculated the correlation coefficients between the three GIS measures of accessibility. All statistical analyses were performed in SPSS Version 11.

## Results

**Figure 2:**
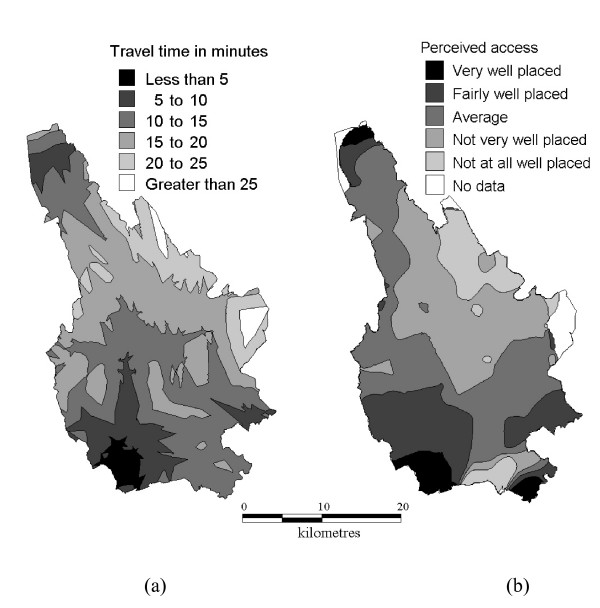
**a) Drive-time to nearest hospital A&E department**. Modelled using Mapinfo Drive-time 6.1 with default travel speeds. **b) Residents' perceived access to hospital A&E departments.** Contours derived from interpolated surface using Vertical Mapper 3.0 with inverse distance weighting, cell size 100 m, search radius 3 km, exponent 2, maximum points 500. Crown copyright material is reproduced with the permission of the Controller of HMSO and the Queen's Printer for Scotland.

**Figure 3:**
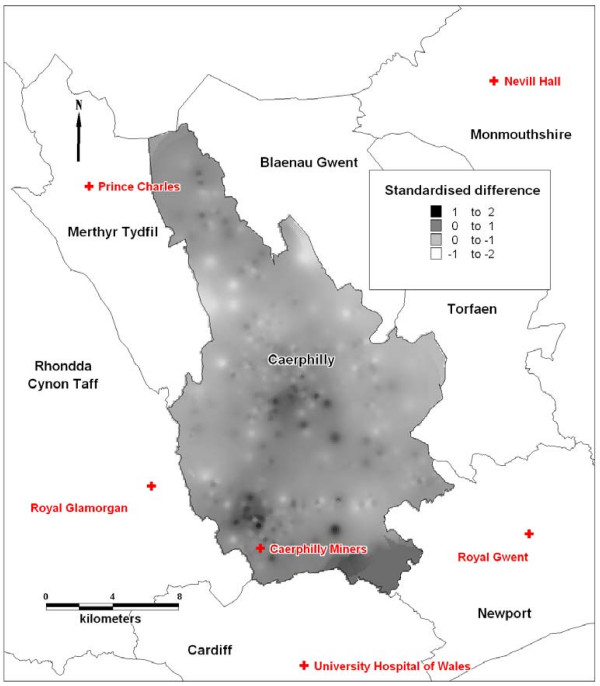
**Map to show standardised differences between residents' perceived access and drive-time to nearest hospital A&E department. **Smoothed using inverse distance weighting, cell size 100 m, search radius 3 km, exponent 2, maximum points 500. The map shows location of the six hospital A&E departments and adjacent county boroughs to Caerphilly county borough. Crown copyright material is reproduced with the permission of the Controller of HMSO and the Queen's Printer for Scotland**.**

Of the 12,408 respondents to the survey, 316 were missing a geographical identifier. Data on a total of 11,853 respondents who answered the question on perceived accessibility to an A&E department with a valid postcode were analysed in this study. The geographical patterns of modelled drive-time and perceived accessibility are shown in Figure 2. The values shown in these figures are not directly comparable, since contours in Figure 2a are lines of equal travel time (travel isochrones), whereas contours in Figure 2b represent interpolated local averages of perceived accessibility. However, the maps appear to show broadly similar patterns of drive-time and perceived accessibility. The overlay map confirms a fairly uniform agreement between perception and modelled drive-time throughout the borough, with an area around Caerphilly Miners hospital and in the central area of the borough where perception was worse than drive-time (Figure 3).

**Figure 4:**
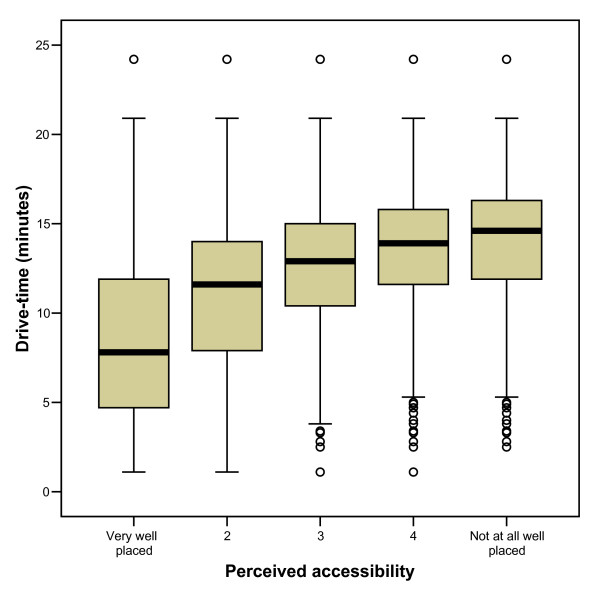
Boxplots of GIS modelled drive-times by categories of perceived access: median, interquartile range, and outlier values.

The overall median drive-time was 12.3 minutes, interquartile range 9.3 to 14.9 minutes. The boxplots of the data show that accessibility based on modelled drive-time generally increased with reducing perceived accessibility but at each level of perceived accessibility there was a wide range of drive-times (Figure 4).

Table 2 shows that drive-time was shorter in residents who perceived better A&E access, better public transport access, and who lived in census output areas with the highest proportion of car ownership. Drive-time varied little by household income or injury status. The Kruskal-Wallis analyses of variance showed significant differences in drive-time by the level of perceived accessibility to A&E, perceived public transport access, and car ownership (p < 0.001). The overall Spearman's rank correlation coefficient for the association between perceived accessibility of A&E departments and modelled drive-time was r= 0.38. This correlation varied by age, but not by gender. Correlation coefficients were highest among respondents aged 45 – 54 years (r = 0.42) and 55 – 64 years (r = 0.45) and were lower than average in age groups younger than 45 years and older than 65 years.

**Table 2:** Modelled drive-time and correlation coefficients for associations with perceived accessibility

**Variable**	**Variable categories**	**N**	**Median drive-time (interquartile range) in minutes**	**Rank correlation coefficient r**
Perceived hospital accessibility	1 (best perceived access)	1590	7.9 (4.7–11.8)	-
	2	2695	11.1 (8.1–14.0)	-
	3	3226	12.6 (10.0–15.1)	-
	3	3226	12.6 (10.0–15.1)	-
	4	2474	13.9 (11.2–15.8)	-
	5 (poorest perceived access)	1868	14.6 (11.8–16.4)	-
				
All respondents		11853	12.6 (9.3–14.9)	0.38
	Male	5270		0.38
	Female	6583		0.38
				
Age (years)	18–24	832	12.6 (9.3–14.9)	0.36
	25–34	1686		0.32
	35–44	2078		0.38
	45–54	2303		0.42
	55–64	2040		0.45
	65–74	1738		0.36
	75+	1176		0.38
				
Household income (annual)	Low	1207	12.4 (9.3–15.0)	0.39
	Medium	4499	12.6 (9.3–15.0)	0.37
	High	5280	11.9 (9.1–14.8)	0.41
				
No car quintile	1 (lowest proportion with no car)	2212	11.2 (7.3–14.6)	0.47
	2	2723	12.0 (9.3–15.0)	0.39
	3	2600	12.6 (10.0–14.9)	0.34
	4	2424	13.9 (11.6–15.5)	0.33
	5 (highest proportion with no car)	2133	12.3 (9.3–14.7)	0.39
				
Perceived public transport access	1 (very well placed)	5140	11.7 (8.5–14.7)	0.41
	2	3114	12.4 (9.3–14.9)	0.37
	3	2204	13.0 (10.3–15.3)	0.31
	4	744	13.3 (10.7–15.7)	0.31
	5 (not at all well placed)	472	12.6 (11.4–16.4)	0.25
Injury	Yes	828	12.3 (9.3–15.0)	0.46
	No	10830	12.3 (9.3–14.9)	0.38

All p-values for the rank correlation coefficients were p < 0.01

Correlation coefficients also varied across the quintiles of household car ownership in a non-linear relation. The strongest correlation was for respondents in areas with the lowest proportion of households without a car. Respondents with best access to public transport had a stronger correlation between perceived and modelled access to A&E (r = 0.41) than did those with poorest access to public transport (r = 0.25), with a clear gradient in correlations across the categories of perceived public transport. Correlation coefficients were similar within each band of household income. The perception of accessibility to A&E correlated better with modelled drive-time among respondents who had recently been treated for an injury than among those not reporting any injury treatment. All correlation coefficients were significantly different from zero with p-values less than 0.01.

The overall correlation with the perceived accessibility variable decreased slightly when we repeated the analyses with either road travel distance (r = 0.36) or straight-line distance (r = 0.38) instead of drive-time. However, irrespective of which accessibility indicator we used, the pattern of correlation coefficients across the categories of respondents examined remained very similar. The correlations between the three GIS measures of access were as follows: drive-time and road travel distance, r = 0.62, p < 0.001; drive-time and straight-line distance, r = 0.92, p < 0.001; and road travel distance and straight-line distance, r = 0.69, p < 0.001.

## Discussion

We found that respondents' perception of accessibility of A&E departments correlated significantly with the accessibility model based on drive-time, road distance, or straight-line distance, although all correlation coefficients were less than 0.5. Correlations were higher among residents of areas where most households have cars and who are therefore less likely to be dependent on public transport. However, the increase in the strength of the correlation between perceived and modelled accessibility to A&E with increasing perceived public transport access suggests how important public transport is for many residents who wish to access health services. The finding that respondents who had been treated for an injury in the last three months had more positive perceptions of accessibility compared to those who had not been treated also suggests that the experience of accessing the health system for injury treatment changes patients' perception of the accessibility.

### Study limitations

Our analysis was limited to an area in which the maximum distance of any resident from an A&E department was about 22 km. This is a relatively small range of accessibility, given that some locations in other parts of Wales are as much as 70 km from a hospital with an A&E department. Our analysis within a narrow range of the distribution of accessibility might not accurately reflect correlations within a wider population. As with all GIS models of accessibility, the data on perceived accessibility are two-dimensional with no attempt to take account of variation over time. Perceptions may well vary over time in response to factors that affect whole populations, such as hospital closure or public transport network change and factors that affect only some individuals, such as change in mobility brought about by illness or disability. The drive-time model does not include public transport journeys and our results support the need for better, integrated measures of geographical access that include both private and public transport in drive-time models [12].

The perception of geographical accessibility of healthcare services is likely to be influenced by geographical factors, such as changes in the geographical configuration of health care services and changes in the public transport network, and non-geographical factors such as individual expectation of need for care and knowledge of service locations. The data that we have used in this study as a measure of perceived spatial accessibility were obtained as answers to a survey question that may not have been interpreted by all respondents as a question about spatial accessibility. Access to health care services is a complex concept [21]. Respondents might have had in mind non-spatial accessibility factors, such as financial considerations, and measures of acceptability of services, such as waiting times or opening hours, in addition to purely geographical factors. This could explain why access was perceived to be worse than modelled travel time in some areas of the borough, but further research is needed to investigate the importance of these other factors.

We used the default travel speed matrix supplied with the software as it was outside the scope of this study to validate these locally. As with all drive-time analyses, no explicit adjustment could be made for a host of factors that will influence drive-time, including peak or off-peak journeys, the time of year and weather conditions, or the type of vehicle. We were unable to perform a traditional sensitivity analysis because although the drive-times would change, the absolute value of the modelled speeds does not affect the patterns of correlation. It would also have been interesting to have asked a question on perceived accessibility in quantitative time and distance to compare with the categorical responses and therefore investigate whether respondents generally over- or under-estimated travel time compared with modelled drive-time.

### Contribution to the literature

To our knowledge, no other published papers include a measure of perception of access to A&E departments or hospitals more generally, in the UK or internationally. Indeed it has been suggested that actual and perceived accessibility to hospital should be investigated and compared [8]. Our study adds to previous studies of geographical access to primary care services [7][9][12] and hospital services [4][5,7-9,11,12] which have demonstrated the utility of GIS drive-time modelling.

Drive-time, which allows for variation in travelling speed on different roads, is usually regarded as a more sophisticated measure of geographical separation than travel distance, which in turn is regarded as a more sophisticated measure than straight-line distance. However, in our analysis, replacing drive-time with less sophisticated measures only slightly changed the overall correlation with perceived access. In line with studies of access to primary and secondary care services in south-west England, we also found a strong correlation between straight-line distance and drive-time [9,12]. These studies found the correlation was weaker in rural and peripheral areas, but we were unable to investigate urban-rural differences in our study which was based within a predominately urban setting. Our results suggest that in studies that aim to model geographical separation in a way that correlates well with the perception of patients and potential patients, there may be minimal advantage in using sophisticated measures. Straight-line distance, which can be calculated without a GIS, may be as good as GIS-modelled drive-time or distance for this purpose.

We cannot assume that either perceived accessibility or GIS models of accessibility are good predictors of the actual travel experiences of patients. There is, for example, evidence that for hospital-based haemodialysis treatment, travel experiences of patients in Wales are substantially more complex than could be predicted by any model that assumes attendance at the nearest service point and travel via the shortest or quickest route [22]. However, despite some attempts to take account of complex travel patterns, conventional drive-time and distance models are likely to remain the most commonly used approach to investigating spatial accessibility of health care services for the foreseeable future.

## Conclusion

Although travel experiences of patients will be more complex than could be predicted by any GIS model that assumes both attendance at the nearest hospital and travel via the shortest or quickest route, this study provides evidence that the accessibility of hospital-based health care services as perceived by local residents is significantly correlated to measures of spatial accessibility modelled using GIS. The results of this study will be of importance to health policy makers and local planners, particularly in the context of Accessibility Planning in the NHS which aims to "ensure that there is clearer, more systematic approach to identifying and tackling the barriers that prevent people, especially those from disadvantaged areas, accessing the jobs and key services that they need". To achieve this aim, Accessibility Planning requires local information on accessibility to services, through focussed assessments of local residents concerns over accessibility, which will include surveys, public consultation, review of research projects and GIS modelling [2]. This study shows that a thorough local assessment can be achieved. We have shown how to collect representative and robust information on residents' perceptions of access to local A&E services and some features of GIS modelling that can develop a greater understanding of local accessibility. Our approach is reproducible and generalisable throughout the UK and internationally and helps to clarify the importance of collecting robust local information on how individuals perceive access to services.

## Competing interests

The author(s) declare that they have no competing interests.

## Authors' contributions

DF is the principal investigator of the Caerphilly Health & Social Needs Study. SC and NL made a substantial contribution to running the survey. DF and SC jointly designed this geographical study. SC and NL carried out the GIS analyses. DF and SC carried out the statistical analyses. All authors jointly drafted and critically revised the paper, and read and approved the final manuscript.

## References

[B1] Department of Health (2004). The NHS Improvement Plan – Putting People at the Heart of Public Services.

[B2] Department of Health (2004). Accessibility planning – an introduction for the NHS.

[B3] Phibbs CS, Luft HS (1995). Correlation of travel time on roads versus straight line distance. Med Care Res Rev.

[B4] Martin D, Roderick P, Diamond I, Clements S, Stone N (1998). Geographical aspects of the uptake of renal replacement therapy in England. Int J Population Geography.

[B5] Roderick P, Clements S, Stone N, Martin D, Diamond I (1999). What determines geographical variation in rates of acceptance onto renal replacement therapy in England?. J Health Serv Res Policy.

[B6] Haynes R, Bentham G, Lovett A, Gale S (1999). Effects of distance to hospital and GP surgery on hospital inpatient episodes, controlling for needs and provision. Soc Sci Med.

[B7] Lovett A, Haynes R, Sunnenberg G, Gale S (2002). Car travel time and accessibility by bus to general practitioner services: a study using patient registers and GIS. Soc Sci Med.

[B8] Brabyn L, Skelly C (2002). Modelling population access to New Zealand public hospitals. Int J Health Geogr.

[B9] Martin D, Wrigley H, Barnett S, Roderick P (2002). Increasing the sophistication of access measurement in a rural healthcare study. Health Place.

[B10] Wood DJ, Gatrell AC (2002). Equity of geographical access to inpatient hospice care within North West England: A Geographical Information Systems (GIS) approach.

[B11] Christie S, Fone D (2003). Equity of access to tertiary hospitals in Wales: a travel time analysis. J Public Health.

[B12] Jordan H, Roderick P, Martin D, Barnett S (2004). Distance, rurality and the need for care: access to health services in south west England. Int J Health Geogr.

[B13] Guagliardo MF (2004). Spatial accessibility of primary care: concepts, methods and challenges. Int J Health Geogr.

[B14] Lyons RA, Lo SV, Heaven M, Littlepage BNC (1995). Injury surveillance in children – usefulness of a centralised database of accident and emergency attendances. Inj Prev.

[B15] Farmer J, Hinds K, Richards H, Godden D (2004). Access, satisfaction and expectations: a comparison of attitudes to health care in rural and urban Scotland.

[B16] Afilalo J, Marinovich A, Afilalo M, Colacone A, Léger R, Unger B, Giguère C (2004). Nonurgent emergency department patient characteristics and barriers to primary care. Acad Emerg Med.

[B17] Fone DL, Jones A, Watkins J, Lester N, Cole J, Thomas G, Webber M, Coyle E (2002). Using local authority data for action on health inequalities: the Caerphilly Health and Social Needs Study. Br J Gen Pract.

[B18] Glennerster H, Lupton R, Noden P, Power A (1999). Poverty, Social Exclusion and Neighbourhood: Studying the area basis of social exclusion. CASE paper 22.

[B19] Office for National Statistics (2004). Percentage of people whose income is below various fractions of median income. Social Trends 34.

[B20] Office for National Statistics (2004). Wales: its people. Living standards.

[B21] Gulliford M, Figueroa-Munoz J, Morgan M, Hughes D, Gibson B, Beech R, Hudson M (2002). What does 'access to health care' mean?. J Health Serv Res Policy.

[B22] Christie S, Morgan G, Heaven M, Sandifer Q, Woerden H (2005). Analysis of renal service provision in south and mid Wales. Public Health.

